# Current concept: personalized alignment total knee arthroplasty as a contrast to classical mechanical alignment total knee arthroplasty

**DOI:** 10.1186/s42836-024-00246-2

**Published:** 2024-05-06

**Authors:** Takafumi Hiranaka

**Affiliations:** Department of Orthopaedic Surgery and Joint Surgery Centre, Takatsuki General Hospital, Osaka, 569-1192 Japan

**Keywords:** Knee, Arthroplasty, Personalized, Kinematic, Alignment

## Abstract

Mechanical alignment (MA) total knee arthroplasty (TKA), with neutral leg alignment, mechanical component alignment, and parallel gaps, has achieved good long-term survival. Patient satisfaction, however, is not always perfect. In contrast to the MA, which aims for an ideal goal for all patients, an alternative has been proposed: kinematic alignment (KA)-TKA. In KA, the articular surface is replicated using components aligning with the three kinematic axes. KA-TKA has been gaining popularity, and in addition to the true or calipered KA, various derivatives, such as restricted KA, soft-tissue respecting KA, and functional alignments, have been introduced. Moreover, the functional approach encompasses several sub-approaches. This somewhat complicated scenario has led to some confusion. Therefore, the terminology needs to be re-organized. The term “personalized alignment (PA)” has been used in contrast to the MA approach, including all approaches other than MA. The term “PA-TKA” should be used comprehensively instead of KA and it represents the recent trends in distinct and unique consideration of each individual case. In addition to a comparison between MA and KA, we suggest that evaluation should be conducted to decide which approach is the best for an individual patient within the “personalized alignment” concept.

## Introduction

Mechanical-alignment (MA) total knee arthroplasty (TKA), which features neutral leg alignment, mechanical component alignment, and parallel gaps, has achieved good long-term survival [1–3]. However, patient satisfaction is still imperfect. Kinematic alignment (KA) TKA has been proposed as an alternative: the articular surface is replicated using the components with the three kinematic axes in alignment. Owing to its popularity, in addition to the true or calipered version, some derivatives have been introduced, such as restricted KA, soft-tissue respecting KA and this has created some confusion in the interpretation of the meaning of KA-TKA. Moreover, the recently introduced functional alignment, which aims for perfect gap-balancing, instead of resurfacing in KA-TKA, is considered as a kind of KA, causing a confusion in interpretation. Therefore, it is more appropriate to use the term “personalized alignment” (PA), which aims for the patient’s individual goal and includes all approaches other than MA, as a term in contrast to MA. Thus PA-TKA includes all approaches other than MA, aiming for the best goal individually. In addition to comparison between MA and KA, we suggest evaluation should be conducted to decide which PA approach is the best for an individual patient.

## Mechanical alignment concept

Good long-term survival and clinical outcomes have been attained with MA because of sophisticated operating procedures and improved components [2–5]. The success is attributed to a systematic strategy aiming at a single goal, so-called (neutral) mechanical alignment (MA). In MA, the goal is a neutral leg alignment (hip-knee-ankle angle [HKA] = 0°), mechanical component alignment (components are perpendicular to the mechanical axis) and balanced gap (flexion and extension gaps are the same and parallel) [1]. Since mechanical stability and balance are prioritized in this approach, to some extent, the characteristics of individual patients can be overlooked.

However, leg alignment is not straight but actually slightly varus, and constitutional varus (HKA < -3°) is prevalent [6]. This propensity is especially obvious in Asian countries [7, 8]. In such alignment, the distal femoral and proximal tibial cutting surfaces are not parallel to each other, creating a scenario in which the surgeon must decide whether to release the soft tissue, to accept or to recut. According to the concept of MA, soft tissue release is necessary to attain neutral alignment, but it can cause instability, imbalance and/or pain [9]. Recently, the medial preserving gap technique, in which the medial gap is precisely adjusted, with lateral laxity being accepted, and has been reported as an “accept” option [10]. As for the “recut” option, the overall leg alignment would not be changed, but it will no longer be a “true” mechanical alignment. Instead, it becomes a kind of personalized alignment because not a neutral alignment is created.

Regarding the joint line, it inclines medially by 3° or 4°. The inclination varies with various patients, and the joint line obliquity (JLO) itself reflects the characteristics of individual knees [11]. Despite the variation in joint line obliquity, the bone resection is always perpendicular to the mechanical axis, so, to some extent, joint line will be altered. Although there are balanced extension and flexion gaps, this can create mid-flexion instability [12]. As for ligament balancing, the lateral gap is normally looser than a medial one, especially in flexion [13]. A slight tighter medial gap is considered advantageous for medial pivot motion.

## KA-TKA

In contrast to the systematic MA approach, a new concept has emerged with respect to the patient-specific alignment and appropriate placement of the prosthesis. It began with the custom-fit TKA, which was reported by Howell et al. in 2008, [14] and subsequently evolved into the well-known KA-TKA concept [15, 16]. KA-TKA aims to restore the patient’s original articular surface using artificial components. This approach can be deemed an ultimate style of classical anatomical approach [17] because complete resurfacing is believed to be impossible under the mechanical alignment concept as the individual patient has its own leg alignment and joint line obliquity which are not always neutral [6, 11]. The goal of alignment thus differs with various patients. Initially, it was performed as a type of computer-assisted surgery called “patient-specific instrumentation” [14]. Recently, KA-TKA has been performed using manual instruments (calipered KA) [18]. The calipered KA, also known as “true KA”, “pure KA”, “unrestricted KA”, involves osteotomies parallel to the articular surface of both the femur and the tibia, compensating for cartilage wear. The gap is balanced, and soft tissue release is rarely necessary. The KA concept is completely different from its MA counterpart, which emphasizes mechanical stability, and the components would not be set perpendicular to the mechanical axes. As a result, a concern emerged that component alignment could be an outlier in terms of the MA concept, and the mechanical stability may be negatively affected [19]. However, good long-term results have been reported in recent years, comparable with those of MA-TKA for up to 10 years [20]. Another report of a large number of patients from two national joint registries showed a similarity between the revision rates of unrestricted KA and all other types of TKA [21].

## Derivatives of KA-TKA

The popularity of the KA-TKA technique has led to some derivatives. Despite increasing evidence of satisfactory results from unrestricted KA, extreme alignment of the whole leg and components remain a concern [19]. Restricted KA (rKA), where the osteotomy of the KA concept is performed within a safe range, e.g., 3°–5°, has been introduced to address these concerns [22–24]. Although the osteotomy is done at a defined angle in patients with alignment out of the safe range, good soft tissue balancing can be established, and satisfactory mid-term results have been reported [23, 25]. However, the rKA approach can only be precisely accomplished with computer-aided navigation and patient-specific instrumentation, which means it cannot be performed in all centers.

Another KA approach has been proposed by a small number of authors. With this approach, the tibial cut is made referring to soft tissue balance. After the femur is cut, leg is pulled distally (in-line traction) and a line is drawn on the anterior surface of the tibia parallel to the femoral distal cutting surface [26]. In another technique, after completing the femoral cut with the KA philosophy, the residual medial gap is measured using sizing gauges, and the medial slope of the tibial cut is decided with consideration of the medial gap [27]. One recent paper detailed the procedure and its initial results [28]. This technique assumes that the femoral and tibial joint lines are parallel to each other and that the gap is balanced in extension in a normal knee.

In addition, the use of surgical robots and navigation systems, which have recently become widespread, provides intraoperative information on bone cutting thickness, soft tissue balance, and alignment of the global leg and components. Using this attribute of robotics, the functional alignment (FA) technique has been emerged, in which components are placed in the optimal position to attain the balanced gap throughout the knee arc by manipulating the femoral and tibial cutting planes [29, 30]. The details are described in the following section.

## Functional alignment

The functional alignment aims to generate a balanced gap throughout the knee arc. This approach has been executed according to intraoperative planning based on the data regarding gap, alignment and bone cut thickness provided by modern computer-aided devices such as navigation or robotics [29–32]. This is considered to be an ultimate style of the gap-balancing technique and the classical functional approach [17]. The deliveries arise from the base of the gap creation. First with the original functional approach, the femoral and tibial cutting planes are adjusted simultaneously to achieve the balanced gap [29]. Within the functional approach, there are two adjustment strategies; one is the mechanical start, where the manipulation is started from the mechanical position (perpendicular to the mechanical axes); the other is kinematic start, which start from the kinematic position (parallel to the joint line). In fact, the two approaches seek to achieve a similar goal [32]. Second, in the tibia-based approach, known as the inverse kinematic approach [33], and the tibia is cut first to simulate the tibial articular surface, the distal and posterior femoral cutting surfaces are then decided based on the gap provided intraoperatively [33–35]. Third, the femur-based approach, the femur is resected in calipered manner, the distal and posterior end of the femur condyles are resected at the same thickness as the components, compensating for cartilage wear. The tibial plane is manipulated using intraoperative gap data to accomplish the balanced gaps [36, 37].

In contrast to the functional alignment approach, the kinematic alignment approach aims to restore the pre-disease articular surface using artificial components [16, 18]. Both approaches theoretically reach the same goal because the morphology of the articular surface perfectly matches the surrounding soft tissue. However, there are no components that can perfectly replicate the constitutional morphology of individual knees [38]. Moreover, even with perfect replication of the morphology, contracture and/or elongation of surrounding soft-tissues [39, 40] as well as the sacrifice of the anterior cruciate ligament and occasionally the posterior cruciate ligament can cause divergence between them [41, 42]. Some modification of component position is therefore necessary if the soft-tissue imbalance cannot be ignored [43]. Conversely, the functional approach can totally achieve the proper soft tissue balancing, but there is an inevitable alteration of the articular surface. Although the extension gap is set to be parallel and lateral laxity has been allowed in most reports [37, 44], the extent to which imbalance is acceptable at respective knee flexion angles has not been established by evidence. Like the alignment boundary for the restricted KA [22], the soft-tissue boundary for the functional alignment should be decided.

## The concept of personalized alignment as a contrast to MA

**Fig. 1:**
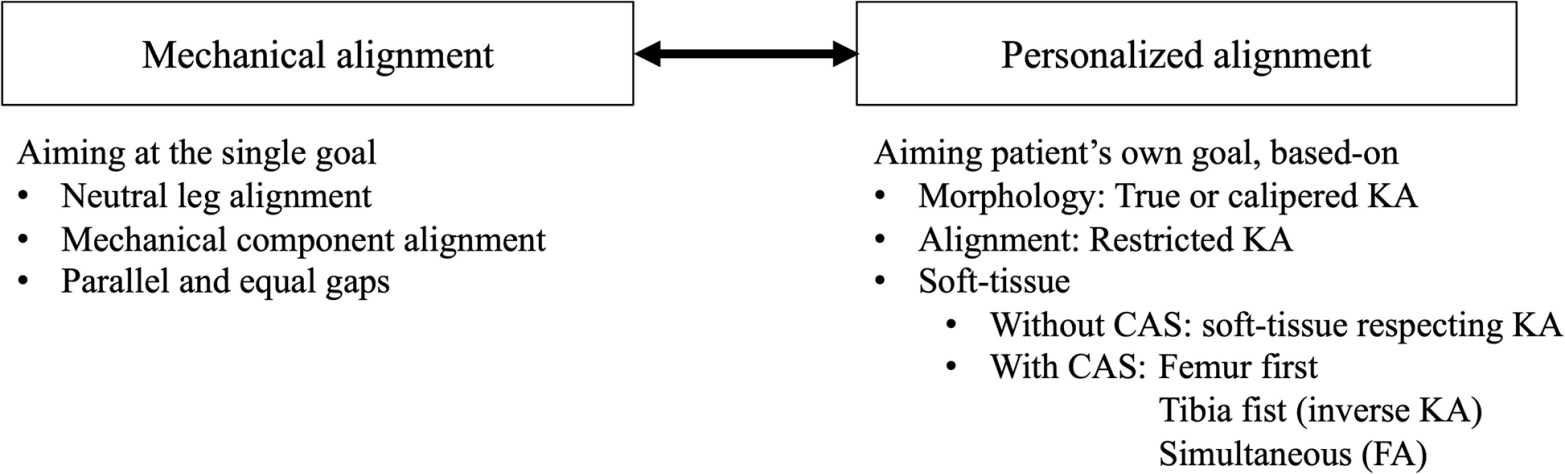
Mechanical alignment and personalized alignment. Personalized alignment is a contrast concept to mechanical alignment. Personalized alignment includes all types of kinematic alignment, functional alignment and their respective deliveries

As various approaches have emerged, the term “KA” may now cause misinterpretation and confusion [45]. Not only the choice between MA or KA, but also the specific type of KA is important, and outcomes should be compared between the KA approaches. This has resulted in the term “personalized alignment (PA)”, as aforementioned [46]. PA includes the true KA and its derivatives and functional alignment. It contrasts with the concept of MA, where all surgeries aim have the single goal of neutral-mechanical alignment. With the PA concept, the goal differs with patients, so it is “personalized”. KA, as well as FA, is thus considered to be a kind of PA [46]. Moreover, in terms of the goal being personalized and a strict neutral-mechanical alignment not being the aim, even an adjusted mechanical alignment can be included within the PA concept (Fig. 1).

**Fig. 2:**
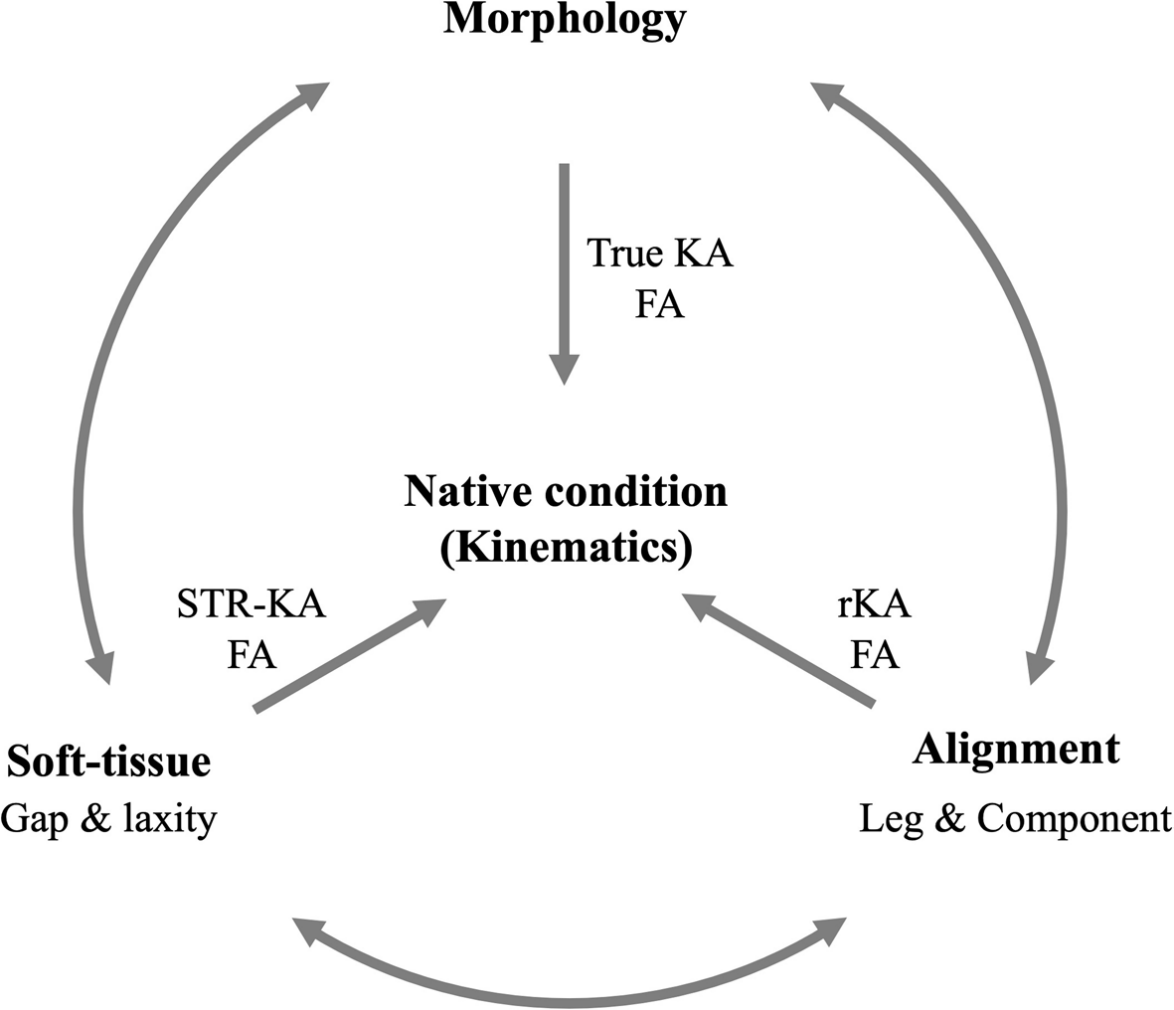
Three knee element theory that helps understand personalized alignment (PA). Each element (morphology, soft-tissue and alignment) decides and is decided by the others with the best harmony, constructing the patient’s native condition and consequent kinematics. The PA aims to achieve this condition, but there are several approaches and the starting point (priority of the elements) differs with the approaches. KA: kinematic alignment, STA: soft-tissue respecting, FA: functional alignment, rKA: restricted kinematic alignment

For a better understanding of PA, the three-element theory (morphology, alignment and soft tissue) of the knee is useful (Fig. 2) [45, 47]. The best harmony of these elements is assembled; an element decides and is decided by the others, resulting in its kinematics in individual knees. True KA starts from morphology, rKA starts from alignment, and the soft-tissue respecting approach and functional alignment start from the soft-tissue balance. Theoretically, the same goal will be achieved, but bone and cartilage defects, which cannot be accurately estimated, and soft-tissue abnormalities, such as contracture and elongation, can make the goal ambiguous. In this situation, derivatives are induced by which of the elements should be prioritized and which of them are compromised. However, the rKA approach can only be precisely accomplished with computer-aided navigation and patient-specific instrumentation, which means it cannot be performed in all centers.

## Future of personalized alignment

Several unanswered questions regarding PA remain to be addressed. It is necessary to confirm whether or not restriction is required. If restriction is required, an evidence-based definition of such a boundary is required. Moreover, this boundary may need to be tailored to race, gender, age, and other factors. It is also necessary to verify whether this PA method improves the function and satisfaction of patients. Moreover, it is necessary to examine not only conventional functional evaluation and patient-reported outcome measures, but also more objective measures, such as data from wearable devices. More importantly, which PA approach is best for the patients has to be assessed.

The most discussion has been focusing on the coronal plane alignment. However, sagittal alignment, such as flexion angle of the femoral component and posterior slope of the tibial component may also play an important role [48] and should be a subject of study in the future. Moreover, the existing implants are made for MA approach. When using these implants under approaches other than MA, some problems, especially in the patella-femoral joint, can arise [38]. Although the influence of KA and other PA approaches on the patellofemoral is controversial [49–52], the specific implants for PA should be developed [38].

## Conclusion

In an era of diversity, the individuality of each person is respected. The concept of PA is fully in line with the notion. TKA has shifted toward finding the optimum PA alignment that respects the individual characteristics of each patient. This differs from installation of components to a single set of conditions, the previous goal of MA. Meanwhile, various methods have been proposed, resulting in confusion. In addition to overall comparison between MA and KA, which of the PA approaches is best for individual patients should be evaluated.

## Data Availability

Not applicable.

## Abbreviations

KA: Kinematic alignment
MA: Mechanical alignment
PA: Personalized alignment
rKA: Restricted kinematic alignment
TKA: Total knee arthropasty
